# Traditional Artificial Neural Networks Versus Deep Learning in Optimization of Material Aspects of 3D Printing

**DOI:** 10.3390/ma14247625

**Published:** 2021-12-11

**Authors:** Izabela Rojek, Dariusz Mikołajewski, Piotr Kotlarz, Krzysztof Tyburek, Jakub Kopowski, Ewa Dostatni

**Affiliations:** 1 Institute of Computer Science, Kazimierz Wielki University, 85-064 Bydgoszcz, Poland; dmikolaj@ukw.edu.pl (D.M.); piotrk@ukw.edu.pl (P.K.); krzysiekkt@ukw.edu.pl (K.T.); kopowski@ukw.edu.pl (J.K.); 2 Faculty of Mechanical Engineering, Poznan University of Technology, 60-965 Poznan, Poland; ewa.dostatni@put.poznan.pl

**Keywords:** 3D printing, material, process optimization, artificial neural network, deep learning

## Abstract

3D printing of assistive devices requires optimization of material selection, raw materials formulas, and complex printing processes that have to balance a high number of variable but highly correlated variables. The performance of patient-specific 3D printed solutions is still limited by both the increasing number of available materials with different properties (including multi-material printing) and the large number of process features that need to be optimized. The main purpose of this study is to compare the optimization of 3D printing properties toward the maximum tensile force of an exoskeleton sample based on two different approaches: traditional artificial neural networks (ANNs) and a deep learning (DL) approach based on convolutional neural networks (CNNs). Compared with the results from the traditional ANN approach, optimization based on DL decreased the speed of the calculations by up to 1.5 times with the same print quality, improved the quality, decreased the MSE, and a set of printing parameters not previously determined by trial and error was also identified. The above-mentioned results show that DL is an effective tool with significant potential for wide application in the planning and optimization of material properties in the 3D printing process. Further research is needed to apply low-cost but more computationally efficient solutions to multi-tasking and multi-material additive manufacturing.

## 1. Introduction

Additive manufacturing (3D printing) has been widely used in clinical practice since the 1980s, including, for example, for preoperative simulation, training, and manufacturing of implants and rehabilitation supplies. Process design methodologies and models can be implemented more efficiently and faster using artificial intelligence (AI), including machine learning (ML). The following AI methods and tools are used:

- Determining the structure of the technological process (sequences of technological operations and procedures): decision rules
- Building models of selecting materials, semi-finished products, tooling and their parameters, and settings: artificial neural networks (ANN) and decision trees (DT)
- Pre-processing (normalization, coding) of selected data used to build models: fuzzy logic, including ordered fuzzy numbers (OFN)
- Implementation of models for the selection of materials, semi-finished products, tools, devices, and parameters of their processing as a prototype expert system used to design the technological process: ANN
- Attempts to eliminate disturbances in the course of the planned technological process affecting product quality by means of the developed methodology and models of technological process supervision: process instability, exceeding warning and alarm values of monitored parameters by means of ANN and DT
- Predictive models, including for control and compensation of deformations (including thermal ones): ANN
- Ageing processes: ANN.

An artificially intelligent system is designed to support technologists, both experienced (as an opinion system) and inexperienced (as a system that complements their knowledge and experience, and teaches) in process design. Methodologies, models, and prototype expert systems are developed based on copying the activity of a human who is an expert in a given field, with the ability to gather experience and knowledge, analyze data, and draw conclusions to solve problems. Research conducted within the project will demonstrate their usefulness and effectiveness in the design and supervision of the technological process of 3D printing. In addition, application of AI methods will increase the use of data included in technological databases. Types, structures, and parameters of the learning and testing processes have been optimized to make efficient use of knowledge, including that extracted in real time from sensors. We expect interesting research conclusions and the emergence of models that are more effective than existing ones.

The main purpose of this study was to compare the optimization of 3D printing properties toward the maximum tensile force of an exoskeleton sample based on two different approaches: traditional ANN and a DL approach based on convolutional neural networks (CNNs). The plan is to solve the same technological problems using two completely different tools and compare the results obtained and the effectiveness of both approaches: traditional ANNs and DL. In particular, the idea is to avoid, as far as possible, generating results on a “black box” basis (i.e., without an explanation of how the result was obtained)—in this context, decision trees are more intuitive and simpler than ANNs. This is particularly important in the production of individualized, one-off production, with a large number of product variants, and therefore a low degree of standardization. In this way, knowledge gained from experienced technologists and from already designed and tested technological processes, proven in production, can be effectively used to design new technological processes for new products. This labor-intensive process, requiring many consultations with technologists, can be performed more efficiently, quickly, and accurately, bringing a new quality to CAPP (Computer Aided Process Planning) systems, based in part on technologists’ intuition, which is difficult to describe. The acquisition of new knowledge will also be realized through periodic learning of solutions, i.e., according to incoming new data [1,2,3].

This translates into automatic updates, based on actual data and not just catalogue data, subject to changes over many years. This avoids errors in process design and thus minimizes company losses. The new knowledge gained in the project will significantly improve the implementation of production processes, the optimization of existing technologies and the emergence of new technologies, and is the authors’ contribution to research on artificial intelligence and its applications [1,2,3].

The structure of the article is as follows: we start with theoretical background regarding DL and optimization, then we present the material (analyzed data sets) and research methods/tools (ANN, DL) used in the work. We successively present the results of both approaches, discuss their advantages and disadvantages compared to the solutions of competing teams, and indicate the directions of further research. The work ends with detailed conclusions.

## 2. Theoretical Background

### 2.1. Deep Learning

DL is a ML technique in artificial intelligence that is a rapidly developing area of research and engineering practice. DL far surpasses many of its predecessors in its ability to recognize speech, computer vision, and natural language processing, as well as to implement ML or intelligent machine design. In this paper, we use the deep ML paradigm and different types of neural networks to optimize 3D printing [1,2,3]. In contrast to traditional learning methods, DL refers to ML techniques that use supervised or unsupervised strategies to automatically learn hierarchical representations in deep architectures for the purpose of classifying intelligent patterns. Multilayer information processing in hierarchical architectures is used here for features learning and further learned patterns classification. DL has been combined even more effectively in industrial components that use vast amounts of advanced information. It is at the intersection of the research areas of neural networks, models optimization, pattern recognition, and signal processing. Two main reasons for the popularity of DL are:

- A significant reduction in hardware costs
- Drastically increased computing capabilities of processors (e.g., graphic processing units (GPUs)).

Since 2006, researchers have demonstrated the success of DL in many applications such as computer vision, speech recognition, image feature encoding, semantic classification, handwriting recognition, information retrieval, and robotics [1,2,3].

**Figure 1:**
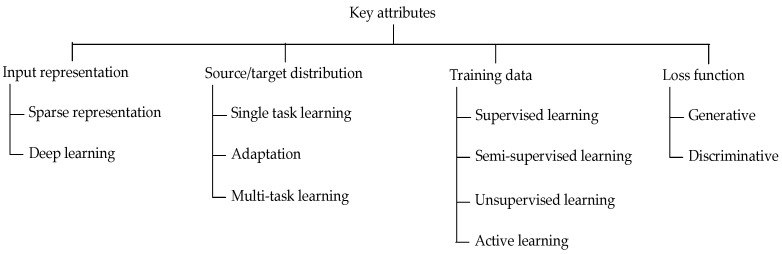
Key attributes used to classify the ML paradigm [1,2,3].

Four key attributes are used to classify a ML paradigm and place it in the context of a specific application: input representation, source and target distribution, training data, and loss function (Figure 1) [1,2,3].

DL can help push the boundaries of what has previously been possible in the field of 3D printing optimization. However, this does not automatically mean that traditional techniques that were gradually developed in the years before DL have become obsolete. It may be that in some applications, legacy solutions will prove to be more effective, and a hybrid approach, combining old and new methods and techniques, will have to be used to solve some problems—these issues still require further research, and this paper is one of the first to address this complex problem [2,3,4,5,6,7,8] Tables S1–S3.

A multilayer perceptron (MLP) is a feed-forward ANN that has a minimum of three layers:

- Input layer
- Hidden layer
- The output layer.

**Figure 2:**
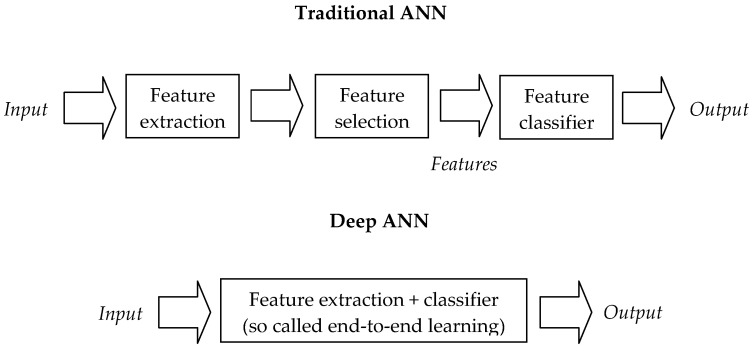
Traditional vs. Deep Learning workflow [4].

The neurons in MLP use a non-linear activation function (Figure 2). The main disadvantage of the MLP is that it has many parameters due to its full internal connection. This can result in redundancy and inefficiency.

**Figure 3:**
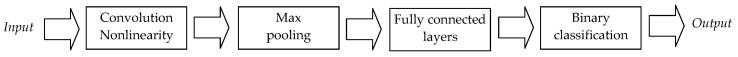
Building blocks of a CNN [4].

CNN is also a feed forward neural network. The core element of CNN’s architecture is the convolution layer, consisting of a set of learning filters. In CNN hidden layers, the convolution, and linking functions are usually used instead of using the normal activation functions (Figure 2 and Figure 3). 

Adaptive process control and sensor fusion can be an important part of smart manufacturing [9]. ML can be divided into:

1. reinforcement learning:
  - Q-learning
  - Deep Q-network
2. Supervised learning:
  - Regression (neural networks, decision trees, ensembles methods, linear, non-linear (GLM logistic)
  - Classification (naive Bayes, k-nearest neighbors–kNN, discriminant analysis, support vector machines—SVM)
3. Unsupervised learning:
  - Autoencoders
  - Clustering (k-means, hierarchical, neural, Gaussian, hidden) [4].

**Figure 4:**
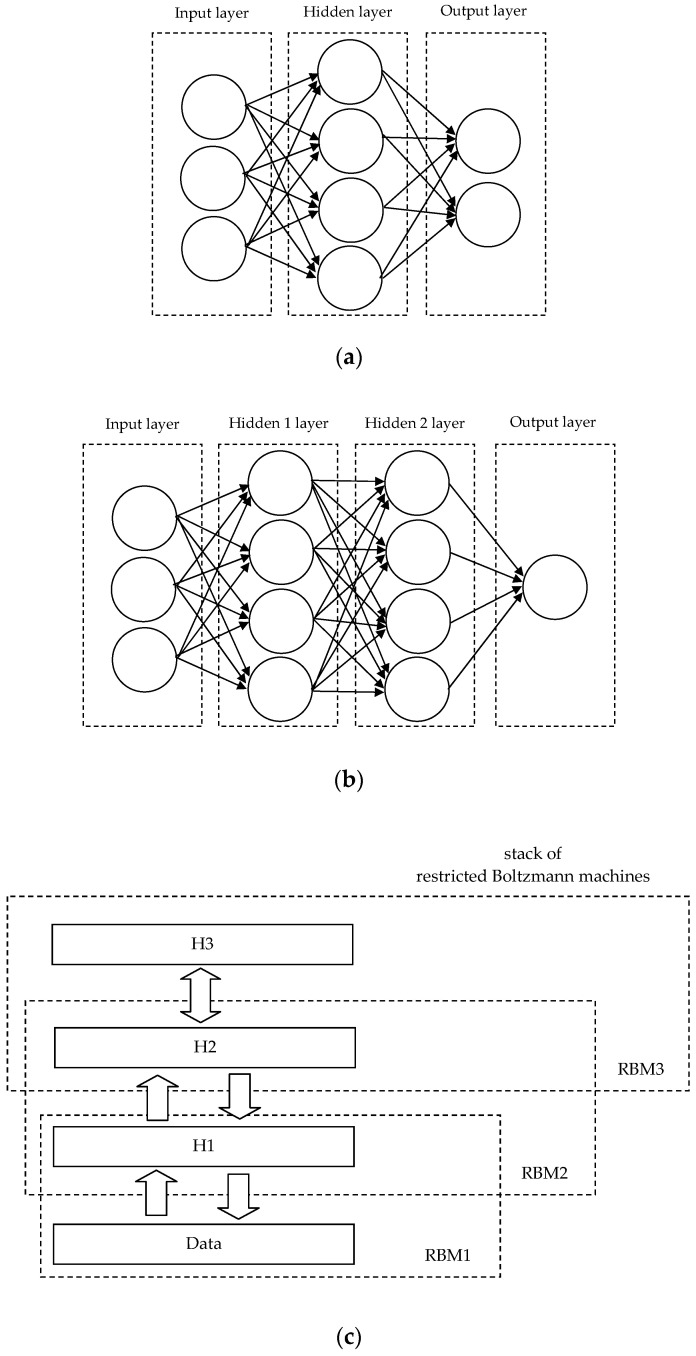
Main architectures: (**a**) ANN, (**b**) CNN, (**c**) DBNN [4].

Main ANN, CNN, DBNN architectures are presented below (Figure 4).

The many methods use various algorithms for implementation, but ANN and SVM are the most popular techniques to implement the ML paradigm. DL is an extended version of supervised learning. CNN and Deep Belief Network are two powerful techniques that can be used to solve various complex problems using DL. DL platforms can also leverage engineering features when learning more complex representations that engineering systems typically do not have. It is absolutely clear that there has been insufficient progress in the development of deep ML systems. One of the most common decision-making tasks in human activity is classification. This classification problem arises when an object must be assigned to a predefined class based on a number of observed attributes associated with that object. Many problems in business, science, industry, and medicine can be treated as such classification problems. Examples include bankruptcy prediction, credit scoring, medical diagnosis, quality control, handwriting recognition, and speech recognition.

### 2.2. Optimization of Solutions

3D printing material features, limitations in the fabrication of complex geometries, and processing parameters have significant effects on the performance of 3D-printed parts (and possibly their therapeutic effect), so it is necessary to optimize these parameters which constitute a difficult task. The idea of optimizing 3D printing and its control systems is key for the development of this group of technologies by relying on new 3D printing technologies, the acquisition and processing of control signals, their classification and interpretation, novel mechanical properties of materials (including programmable strength in different directions and ease of disinfection), and automation of their use in 3D printing (including multi-material printing). AI/ML-based tools can be utilized in different simulation environments. The so-called batch production systems allow for quick product creation and easy modification through recipe amendments—the modifications are made by a technologist without the involvement of programmers. Batch production systems are suitable for all applications where there is mixing and thermal, pressure or chemical processing of many components to obtain a finished product, e.g., for the chemical, pharmaceutical, and food industries. The system itself takes care of the availability of equipment and raw materials needed for production by checking the possibility of fulfilling orders, and if this is not possible, it informs the employees. The production process simulator is a complete, virtual model of a factory with an accurately reproduced production process (a so-called digital twin)—a practical implementation of the idea of Industry 4.0. The digital process simulator makes it possible to check the correct functioning of the entire system before it is implemented in the facility. This makes it possible to control barriers, dependencies, and production processes step-by-step without the risk of losses resulting from wasted material, poor product quality or installation damage. This reduces start-up time: even from several weeks to a few days.

## 3. Materials and Methods 

### 3.1. Data Analysis and Computational Model

The main objective of this study was to compare the optimization of 3D printing properties toward the maximum tensile force of an exoskeleton sample based on two different approaches: traditional ANNs and DL based on convolutional neural networks (CNNs). We want to have a discussion on whether the familiarity with classical ANN optimization techniques should be retained and whether and how it is worthwhile to combine the two sides of optimization (traditional ANN and DL).

### 3.2. Analyzed Data Sets

**Figure 5:**
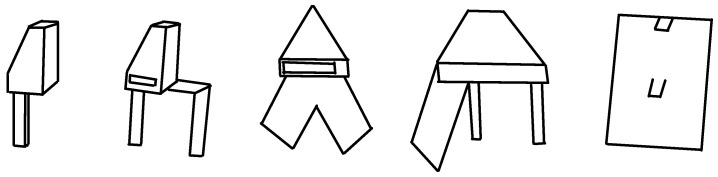
Tested parts of exoskeleton.

For testing of the two computational approaches presented below, five 3D-printed structures (a set of exoskeleton parts of different sizes) were prepared using the FDM technique and checked by the expert for mastering the correctness of the technology and the absence of defects. Examples of the 3D-printed parts are presented in Figure 5.

The Cura 0.1.5 and SLICER software (3D Ultimaker, Utrecht, The Netherlands), and fused filament fabrication (FFF) technology were used in this research to create and 3D print the aforementioned parts of the exoskeleton. Slicing software determined a way to decompose the digital 3D model into layers for printing by an FFF printer. This FFF printer uses a particular sequence of operations to print:

- First, depending on the type of printer, the nozzle, the print bed or both move while the plastic is being extruded
- Simultaneously, the heated nozzle ejects molten plastic, and deposits it in thin layers, one on top of another, layer-by-layer, forming the shape of the whole 3D printed object
- The aforementioned filament layers fuse together due to the thermal fusion bonding occurring between the individual layers, to create a solid part (after cooling down).

To measure the maximum tensile force of the exoskeleton samples, the tests consisted of subjecting each sample mounted in the grips of an INSTRON 5966 testing machine (Instron, High Wycombe, UK) to a monotonically increasing tensile load with a travel speed of the piston of the testing machine of 0.2 mm/s. The tests were carried out at a temperature of 21–23 °C and 55% air humidity. During the test, the instantaneous values of the loading force and displacement of the grip of the testing machine were measured until the sample cracked and completely detached.

Balancing the technical requirements with user safety constraints requires analysis to move from the initial stages of the project.

List of optimized parameters for 3D printing is shown in Table 1.

### 3.3. Testing Procedure

First, the obtained data were analyzed using the ordinary ANN algorithm, and then using DL (CNN), whose task was to enhance the contrast between changes in the 3D print as a result of the material features and identification of selected optimized parameters.

It should be mentioned that a key condition for the replication of our study may be appropriate selection of the used PLA material, its storage, preparation, and then the same procedures with the 3D-printed objects. We are aware that the influence of microstructure and atomic defects on the properties of the materials used and printed objects is assessed as strong.

The data served as the source of the variables for training ANN and CNN, respectively. The above-mentioned data has been divided into two sets: training and testing as follows:

- The training set was used to identify systematic errors and network weights during their learning
- The testing set was used to calibrate, prevent network overtraining, and measure and compare the ANN and CNN performance.

### 3.4. Traditional Approach

To optimize the 3D printing parameters in the traditional way, we used a three-layer feed-forward artificial neural network (ANN) built and trained in the MATLAB environment with Neural Networks Toolbox (version R2021b, MathWorks, Natick, MA, USA). Multi-layer perceptron (MLP) proved to be beneficial to optimize the process parameters in the FFF technique [2]. 

We used:

- Back-propagation (BP) algorithm—a popular gradient-based local search optimization technique
- Naive initialization technique
- Neural network weights preset instead of setting the aforementioned scales to small random numbers to avoid a slow error convergence rate, being trapped at local minima, etc.
- Optimization of the connection weights of the MLP set to minimize the error function (i.e., average mean square error (MSE) between the target and actual outputs averaged over all training examples).

**Figure 6:**
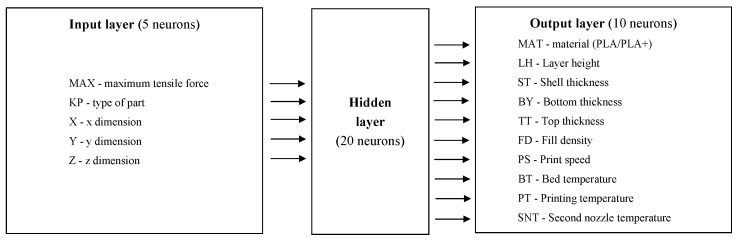
Traditional ANN structure.

The structure of the used ANN is shown in Figure 6 and Table 2.

All of the layers of the ANN contained neurons with the same sigmoid activation function (Table 2).

### 3.5. Deep Learning Approach

To optimize the 3D printing parameters in a deep learning way, we used a four-layer convolutional neural network (CNN) built and trained in the MATLAB environment with Deep Learning Toolbox (version R2021b, MathWorks, Natick, MA, USA).

**Figure 7:**
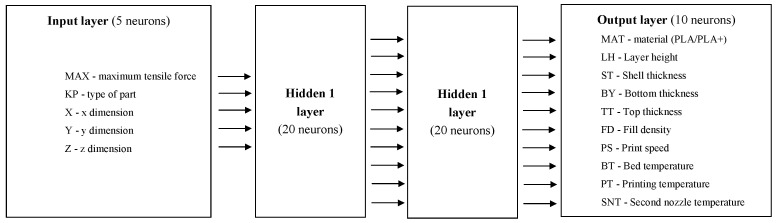
CNN structure.

DL is used in the field of digital data processing to solve problems that are impossible or difficult to solve by traditional CI methods (e.g., detection, classification, segmentation, etc.,), usually with super-human accuracy. We provided here a comparison of simulations on a traditional and deep ANN using the same data in an attempt to answer: from what level of complexity of the system and its description increased calculation effort in deep ANN turns. That is to say: when do we use DL and why? As DL methods, we have used convolutional neural networks (CNNs), which improve the prediction efficiency in most cases by using large amounts of data and abundant computational resources, and push the boundaries of what was possible before, both by humans and traditional CI systems. This is due to the fact that questions have arisen in recent years: does greater use of DL make traditional CI techniques obsolete, or is there still a need to research and develop the study of traditional CI techniques or perhaps even to combine them with DL in the form of hybrid systems? There are still some tasks where traditional CI techniques with global properties are a better solution, especially when considering computing power, time, accuracy, and the characteristics and quantity of the inputs, as far as their application in the Internet of Things (IoT) and mobile solutions is concerned. We compared traditional and deep simulations on the same 3D printing data in an attempt to answer the question: what level of system complexity and its description returns DL’s increased computational effort? The result of the study is to be a suggestion: when should we switch to DL in the optimization of 3D printing and with what calculation processes parameters? The structure of the above-mentioned network is shown in Figure 7 and Table 3.

Almost all layers of the network contained neurons with the same sigmoid activation function, but the Output layer contains linear neurons that provide the easy-to-compare cost function: Gaussian cross-entropy (MSE) (Table 3).

Selection of the functions performed by the hidden layers in the CNN is key for the course of the learning process. The following are possible problems with restricted functions (sigmoidal and hyperbolic tangent) in the hidden layer: unstable gradient, thus learning can get stuck when the feature is saturated. 

## 4. Results

**Figure 8:**
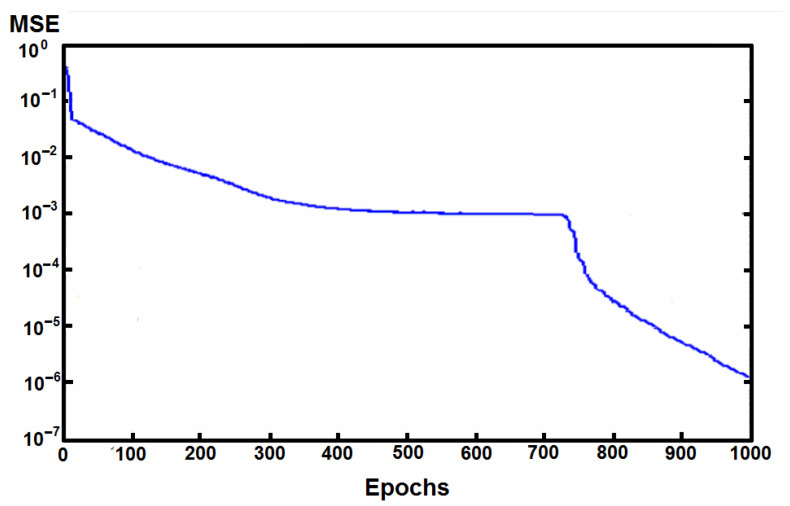
Values of MSE during learning for traditional ANN.

**Figure 9:**
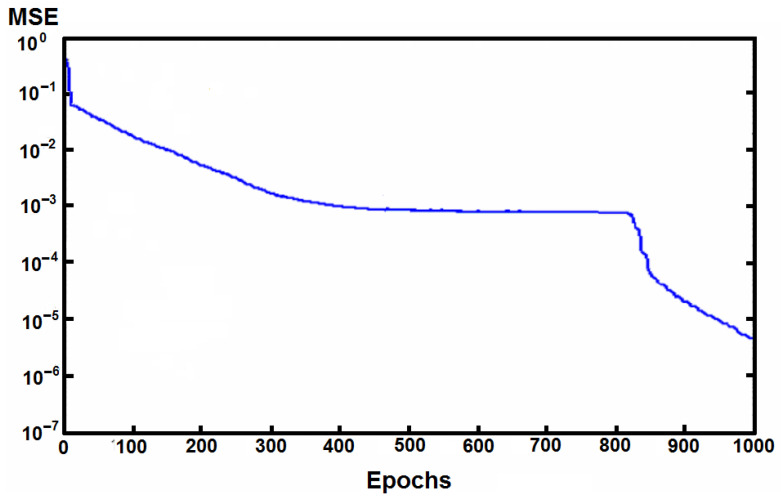
Values of MSE during learning for CNN.

After training and testing the ANN and CNN networks, the results, i.e., the classification accuracy and (R)MSE coefficients, showed that traditional ANN was able to minimize the MSE for the data in the training set to very small values (0.01), made it quicker than CNN, but with lower exactness (Figure 8 and Figure 9, Table 4 and Table 5).

The (R)MSE value as a function of the number of epochs decreased faster in the conventional ANN network (Figure 8).

Compared with the results from the traditional ANN approach, optimization based on DL decreased the speed of the calculations by up to 1.5 times with the same print quality, increased quality (both learning and testing), and decreased MSE, and unique formulas and printing parameters not found previously through trial-and-error approaches were also identified. The longer computation time is the result of the more complex CNN structure (Table 4 and Table 5). Our results indicate that DL is an effective tool with the potential for broad application for planning and optimizing of materials features in 3D printing. CNN has the potential to solve more complex computational tasks; thus, the DL algorithm. It can more quickly predict the behavior of complex physical systems using sparse data sets through integration of physical modeling. The aforementioned shorter time and properties may be increasingly important in the future when it will be necessary to apply the most powerful computational solutions to the most complicated 3D printing projects for their optimization.

Higher values of the quality (learning) and quality (testing) observed in CNN (Table 4) reflected CNN’s better ability to infer from the collected data for the training and testing sets.

**Figure 10:**
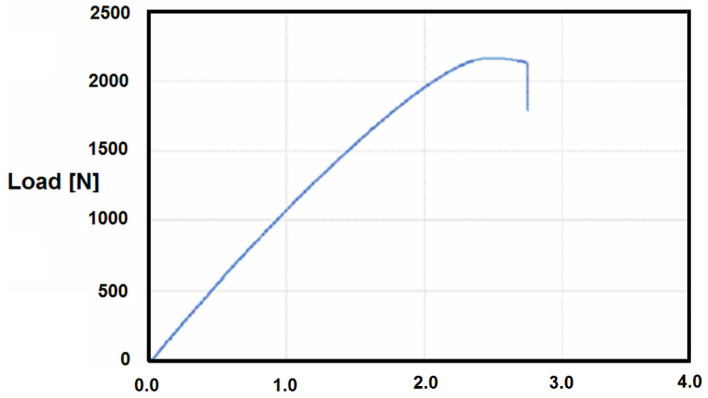
Optimal tensile force of the selected exoskeleton part.

The resultant optimized 3D of the ten printing features established owing to the CNN-based analysis are presented in Table 6, and the optimal tensile force of the selected exoskeleton part can be seen in Figure 10.

Optimal tensile forces estimated at 2122.2 N can practically be compared only to the hand grip strength in the exoskeleton, estimated at 20–60 N, while the grip strength of the ill person may be 50% lower.

In the 3D-printed exoskeleton, material considerations are very important to the design, safety, and usability of the exoskeleton. Optimization requires balancing the many features of the exoskeleton, but AI support can play a key role in this, making the process easier and faster, increasing production efficiency, and the convenience and safety of the end product. 

## 5. Discussion

Our results indicate that the proposed data analysis method is highly effective for optimizing sample parameters, regardless of their shape and size (including depth). Applications of 3D printing have significantly increased in recent years, its broad application in health care is still in progress, especially accompanied by novel AI-based optimization. The use of DL in medical 3D printing parameter selection systems is not obvious, and in rehabilitation engineering, it is not common. Applications of DL in medical science and clinical practice using 3D printing are cited below for comparison. 

Our results confirmed that 3D printing with FFF technology based on the existing PLA/PLA+ material can be optimized for the effective printing of the usable/functional part of the exoskeleton and its strength parameters. 3D printing of exoskeleton elements, and in general, 3D printing for biomedical purposes, is already a complicated issue, because the materials and ready-made elements used, in addition to the specified mechanical and chemical properties, should be biocompatible. It seems that the direction of further research has already confirmed the concept of artificial and intelligent material optimization of 3D printing for biomedical purposes by developing specialized filaments adapted to professional biomedical applications, and then filaments with designed properties corresponding to the needs of the body, its use in combination with living tissue, body fluids, etc. 

In addition, the coexistence of technologies and materials in 3D printing makes it possible to relatively quickly and cheaply produce polymer, metal, ceramic, and even composite/multi-material objects, which are often impossible or too expensive to produce using conventional manufacturing technologies, with unique mechanical, thermal, and dimensional properties. 

The advantage of the proposed research is a holistic approach, not only regarding the selection of the material for 3D printing, but also the choice of technology and taking into account the requirements of a medical device (patient, therapy, therapist). This approach can be a good starting point for building the entire environment connecting software for designing medical devices (MDR, ISO 13485), selecting parameters based on pre-programmable templates and analysis of artificially intelligent requirements, and then optimization of printing, fitting or even necessary corrections.

The main limitation of the study is its focus on a specific exoskeleton solution, which is high technology in itself, perhaps unattainable for some scientists trying to replicate. It seems, however, that the proposed solutions can be easily adapted to simpler 3D printed medical solutions, e.g., orthoses.

The gap in the contemporary scientific and professional literature concerns not only the optimization of artificial intelligence in the selection of material properties for 3D printing, but also the entire process of diagnostics, selection, and adjustment of 3D printed rehabilitation equipment as part of personalized medicine. This does not mean a higher effectiveness of healthcare, but this is because any individual approach is difficult to apply on a mass scale, especially to non-homogeneous groups of patients.

This paper contributes to the existing body of literature because this article is only the beginning of a whole series of works devoted to changing the approach to the rehabilitation supply industry as part of Industry 4.0, and maybe even Clinic 4.0, based on the wider use of artificial intelligence, preventive medicine, and personalized medicine. The challenge is multi-screen printing and the programming of the life cycle of medical devices to best serve patients.

Many different variants of processes, technologies, and materials and their improvements must be considered, creating many novel subtechnologies and possibilities in order to select those that provide new, demanding product features while maintaining the accuracy and speed of production, and the quality of the printed object (final product). Materials whose parameters/functions change with the structural parameters, e.g., with depth, i.e., like in living tissue, constitute an additional challenge. 

Hybrid methodologies can help improve 3D printing performance and solve problems that are not suitable for DL. Combining traditional techniques with profound learning may be popular in new areas for which profound learning models have not yet been fully optimized.

The ability of AI-based systems to monitor the state of knowledge and engineering practice, search/generate new solutions (including alternatives to existing ones), assess progress, dynamically modify the characteristics/parameters of design, planning, production, and recycling (including cycle planning) is becoming increasingly important. Sustainable development requires not only taking into consideration monitoring of life cycle of products but also problem solving approach based on accurate sensors for collecting data, aggregation, inference, and prediction for greater accuracy.

DL is quite often used in 3D printing optimization, including medical applications. 3D printing enables the construction of affordable, patient-specific, anatomically accurate physical models which are more convenient and realistic during simulations of complex (neuro)surgical approaches in a safe didactic environment. All stages of the surgical procedure can be simulated: from positioning and exposure to deep microdissection, taking into account the complex anatomy, working angles, and pathoanatomical relationships. Thermoplastic polymers with different properties can be used to reflect the visual and tactile responses of bones, neurological, and vascular tissues [10]. A personalized 3D model can characterize e.g., a patient’s individual thyroid lesions, not only for medical professionals, but also for the patients and their families. This model can be an effective tool to improve patient understanding and satisfaction. A U-Net-based DL architecture and a 3D mesh modeling technique were used to produce a personalized 3D model of a thyroid gland. The average 3D printing time was long: over 4 h for each patient), but the average production price was only USD 4.23 for each patient. The size, location, and anatomical relationships of the tumor and thyroid gland could be represented better and more accurately, and the group of patients receiving personalized 3D printed models showed significant improvements in all four categories: general knowledge, benefits and risks of surgery, and satisfaction. All patients who received their 3D model found it helpful in understanding the disease, surgery, and possible complications, as well as generally satisfying [11]. DL was used to automatically measure the left ventricular (LV) ejection fraction and also to automatically measure the LVEF using two-dimensional echocardiography (2DE) images for different clinical centers, ultrasound machines, and heart disease phenotypes. A U-Net-based DL algorithm (DPS-Net) was used based on 36,890 frames of 2DE taken from 340 patients, and the two-plane Simpson method was applied to calculate the LVEF. The high performance of the DPS-Net in LV detection and LVEF measurement in heart failure with several phenotypes is shown. This was observed in a large dataset, i.e., DPS-Net is highly adaptive across different echocardiographic systems [12]. Computed tomography (CT) image reconstruction of a life-sized 3D-printed chest phantom placement of tissue mimicking inserts was performed using a commercial reconstruction algorithm (HDFoV) and a novel DL-based approach (HDeepFoV). Reconstruction of images outside the field of view of the CT scanner (e.g., in patients with obesity) requires use of extrapolated data. The DL-based algorithm showed much better performance in quantitative assessments based on 3D-printed phantom data, and in qualitative assessments of patient data [13].

Using low-powered AI acceleration chips, CNN also works interactively on mobile devices (even an iPhone 11 Pro), offering real-time performance in mobile headsets, virtual and augmented reality [14].

3D printing has emerged as a potential way to produce general and personalized IUDs. To ensure controlled release of contraceptive hormones, Monte Carlo simulation and DL models based on ANN, could prove effective in developing precise contraceptive delivery systems, improving the quality of life for women worldwide [15]. Automated face recognition technology based on DL has achieved high accuracy in diagnosing various endocrine diseases and genetic syndromes. A CNN-based facial diagnostic system achieved a high accuracy of 97%, and the results of a prospective study demonstrated the application value of this system in Turner syndrome screening are promising [16].

The recent development of 3D printing has taken hold in healthcare and has led to clinical applications from anatomical models, through devices supporting diagnosis, treatment, rehabilitation, and care, to bioink 3D printing. Although much research to date has focused on materials, designs, processes, and products, little attention has been paid to efforts to enable their commercialization and rapid implementation into clinical practice, including addressing important issues such as reproducibility, quality control, and meeting regulatory requirements. Increasing process uniformity, consistent design, development, and manufacturing will require automation and the use of flexible artificial intelligent information systems, standardization of facilities, equipment, and processes in therapeutic and non-therapeutic applications [17]. Automated pathology detection and 3D vertebral reconstructions based on DL-based labeling and vertebral segmentation methods for biomechanical simulation and 3D printing facilitate clinical decision-making, surgical planning, and tissue engineering [18]. The integrated approach addresses materials processing, fabrication of engineering components and structures including: 3D printing, thin-film and multi-layer structures to obtain coupled mechanical and functional properties. DL solutions are trained to extract the elastoplastic properties of metals and alloys from indentation results using multiple datasets to achieve desired levels of accuracy improvement [19]. High levels of engagement in content-intensive subjects can be difficult to achieve. The majority of students considered 3D-printed models of the skeleton and its parts to be a resource that helped them to improve their study habits, achieve greater confidence, and improve their academic performance [20]. Simulation methods are increasingly used to improve medical skills, allowing trainees/practitioners to practice in a risk-free, reproducible environment. To this end, after segmentation of anatomical features using a 3D printer, several realistic 1:1 scale anatomical models can be produced containing all of the relevant structures, including vascular [21].

Careful surgical planning can determine the success or failure of a whole surgical procedure. A full understanding of the complex spatial relationship between the boundaries of a tumor and the surrounding healthy tissues enables accurate surgical planning. The use of 3D printing to produce anatomical models can be introduced into standard clinical practice, but requires incorporation of best practice and description of a workflow and methodology used to standardize affordable, realistic preoperative virtual and physical simulation that is cost-effective [22]. The study group found the 3D-printed model of a cranial fossa significantly more useful compared to the half skull used by the control group [23]. This approach can be accelerated by optical neural networks, combining wavelet optics with DL methods, demonstrating all-optical inference and generalization to subclasses of data. Combining native or designed dispersion of different material systems with a DL-based design strategy, broadband diffractive neural networks will help to design light-matter interactions in 3D, allowing the creation of task-specific optical components (optically deterministic tasks or statistical inference) [24].

The process of using CAD (Computer Aided Design), Pro/Engineer (Pro/E) software, and 3D printing to construct physical products follows three consecutive required steps:

1. 2D drawing
2. 3D construction of the implant
3. 3D printout for physical printing.

Thanks to the integration of clinical imaging, digital templates and 3D printing, the final prints of, for example, implants can be adapted to the needs of an individual patient, both in terms of shape and material properties [25].

AI should be considered as part of a comprehensive set of solutions, linked to comprehensive specialist education, diagnosis, treatment, rehabilitation and care, 3D printing and virtual/augmented reality technologies and telemedicine, including as part of a coherent therapeutic and business model that can be brought to the healthcare market in the future [26].

Even middle school students are already able to tinker in a virtual world using 3D design software and then tinker in the real world using printed parts, fostering staff development in new specialties [27]. 3D printing allows the creation of typical cyber-physical systems for mass customization, not only in rehabilitation, but also, for example, in dentistry. Short “series” and complex shapes make it necessary to compensate for errors, and doing this manually is neither easy nor economical, hence the need for automatic error compensation. For these reasons:

1. We obtain the shape using technologies such as 3D scanning
2. We use 3D DL to train a deep neural network for a specific task (printing an orthosis or a dental crown)—the CNN can learn the deformation function owing to the large amount of data used for training.
3. We verify the performance of the neural network:

- Translation
- Scaling up
- Scaling down
- Rotation.

The accuracy achieved is sufficient with low hardware and software costs [28].

Endoscopic navigation systems look for integration of big data with multimodal information (i.e., from CT scans, magnetic resonance imaging, ultrasound images, and even external trackers) with respect to anatomy/physiology, patient pathology, controlling the movement of medical endoscopes and surgical instruments, and guiding the surgeon’s actions during intervention (including haptic coupling i.e., transferring tissue properties to the endoscope’s cusps). This allows the introduction of new techniques and promising directions for endoscopic navigation, including 3D printing reconstruction and the creation of teaching aids to support medical simulation [29]. These solutions can be integrated, e.g., with microfluidic devices as a new, low-cost, and convenient platform for, e.g., bacterial cell culture, antibiotic sensitivity, using DL-based vision data regression for robust data reporting [30].

Guidelines and ideas for future research constitute an important impact. In our opinion, optimization of the materials used should be a key part of future medical 3D printing. Novel materials and their pre-projected features may be better tailored to the patient’s needs. The most promising direction for further research is computational analysis and optimization of material and energy suitability (taking into account both efficiency and environmental-friendliness criteria) combined with defect detection and classification as part of quality control in line with the Industry 4.0 paradigm. A signal analysis algorithm and a multi-label classifier based on a deep convolutional neural network (DCNN) trained on the results from active infrared thermography (IRT) has already been applied to evaluate the condition of 3D-printed structures [31]. It should be noted that cracks and pores are also common defects in metal parts produced by 3D printing, hence the need for mass defect detection and classification by segmenting images (still and moving—as in a production line for monitoring the 3D printing process in situ) with defects. This is achieved with almost 100% accuracy using a simple CNN model [32]. A review of DL methods in defect detection highlighted:

- The use of ultrasonic testing, filtering, DL, machine vision, and other technologies used to detect defects
- Classification of product defects into categories in different products
- Functions and characteristics of existing equipment used for defect detection, related to high precision, high positioning, fast detection, small objects, complex backgrounds, hidden object detection and object association
- And only then can DL methods be used to optimize production processes to avoid these defects [33].

Research on a data-driven ML model for predicting the performance of polyhydroxyalkanoates (PHAs) yielded an ML model using a deep neural network (DNN) to predict the glass transition temperature (Tg) of PHA homo- and copolymers. The DNN model performed better here than a support vector machine (SVD), the nonlinear ML model and the least absolute shrinkage and selection operator (LASSO), a sparse linear regression model. Compared to the commonly used ML models using quantitative structure-property relationships, this model does not require an explicit descriptor selection step but shows comparable performance [34]. High defect recognition accuracies by deep networks are not uncommon: an image recognition technique based on convolutional neural networks for multiple concrete defect recognition (CMDnet, 1981 types of concrete surface defects) showed a defect detection accuracy of 98.9% [35]. Verifying the usefulness of DNN and statistical modeling in predicting the strength of bone cements with defects resulting from the introduction of contaminants (blood, saline) into the cement at the stage of its preparation may play an important role in the initial, qualitative assessment of the effects of surgery and in limiting errors resulting in the failure to maintain the required mechanical parameters and, consequently, patient dissatisfaction [36]. A concurrent neural network (ConCNN) with different image scales performs better than other approaches, offering 98.89% classification accuracy with a latency of only about 5.58 ms [37]. Deep ML models allow for material- and energy-efficient designs with a lower environmental impact, e.g., for different strength classes, including optimal recycled content with the lowest cost and environmental footprint [38]. DL-based models also perform well for nanocomposites despite their non-linear nature of processing parameters and the difficulty in predicting the desired features using the conventional regression approach [39]. No doubt it is possible to generalize the DL methodology to a more advanced, multi-material analysis [40], thus we encourage other scientists to develop this area of research and industrial practice. Increasingly many new challenges toward the support of 3D printing by AI are posed not only by predictive operations [41,42,43] and process control [44] under the Industry 4.0 paradigm, but also in terms of eco-design [45] related to the policy of sustainable development and protection of our planet’s potential.

According to the newest research and publications, we can see that the current research is leading in the proper direction compared to the newest studies [46,47].

## 6. Conclusions

Additive manufacturing of medical devices, including soft materials, requires optimization of the materials themselves, sometimes printable inks, raw materials formulas, and 3D printing processes that must balance a large number of variable but highly correlated factors. New 3D printing materials and processes may be as important in rehabilitation as technologies such as biosensors, robotic devices, myoelectric control methods, and advances in brain-machine interaction. New 3D printing materials could be the next breakthrough in patient-tailored devices, but should be cost-effective and useful with semi-automated, AI-assisted matching and decision support.

1. Experimental practices are time- and cost-intensive so the application of AI-based optimization may be a quicker and cheaper solution.
2. PLA-based 3D printing can be optimized to successfully print a utility/functional part of an exoskeleton. Optimization powered by AI/ML can play a key role in the 3D printing process, increasing the efficiency and safety of the printed object (end product).
3. The DL-based approach will become the leader in 3D printing optimization as the complexity of the printed objects increases.
4. Compared with the results from the traditional ANN approach, optimization based on DL decreased the calculating speed by up to 1.5 times with the same print quality, increased quality (both learning: 0.9577 and testing: 0.9721), decreased MSE (0.001), and a set of printing parameters not previously determined by trial and error was also identified.
5. With the current complexity and type of computation, there is no need to combine two optimization solutions (traditional ANN and DL).

## Figures and Tables

**Table 1:** Optimized parameters for 3D printing.

Parameter	Unit
Material choice (PLA/PLA+)	-
Layer height	mm
Shell thickness	mm
Bottom thickness	mm
Top thickness	mm
Fill density	%
Print speed	mm/s
Bed temperature	°C
Printing temperature	°C
Second nozzle temperature	°C

**Table 2:** MLP network model for diagnostic measures.

NS	AH	AO
5-20-10	Sigmoid	Sigmoid

where: NS—structure of ANN; AH—activation function in the hidden layer; AO—activation function in the output layer.

**Table 3:** CNN network model for diagnostic measures.

NS	AH1	AH2	AO
5-20-20-10	Sigmoid	Sigmoid	Linear

where: NS—CNN structure; AH1—activation function in hidden layer 1; AH2—activation function in hidden layer 2; AO—activation function in the output layer.

**Table 4:** Selected ANNs quality assessment.

Network Name	Quality (Learning)	QUALITY (Testing)
MLP 5-20-10	0.9471	0.9676
CNN 5-20-20-10	0.9577	0.9721

**Table 5:** (R)MSE values for used neural networks.

Network Name	(R)MSE
MLP 5-18-10	0.01
CNN 5-20-20-10	0.001

**Table 6:** Optimal parameters for 3D printing.

Parameter	Optimal Value
Layer height [mm]	0.2
Shell thickness [mm]	1.2
Bottom thickness [mm]	2
Top thickness [mm]	2
Fill density [%]	40
Print speed [mm/s]	70
Bed temperature [°C]	55
Printing temperature [°C]	215
Second nozzle temperature [°C]	220
Maximum tensile force [N]	2112.2

## Data Availability

Data sharing is not applicable to this article.

## Supplementary Materials

The following are available online at www.mdpi.com/article/10.3390/ma14247625/s1, Table S1: Main traditional CI technologies, Table S2: Key challenges of CI technologies within 3D printing, Table S3: Traditional ANNs vs. DL.
