# Supplementary material for: Traditional Artificial Neural Networks Versus Deep Learning in Optimization of Material Aspects of 3D Printing
Source: Materials (Basel). 2021 Dec 11;14(24):7625. doi: 10.3390/ma14247625 (PMC8707385; doi:10.3390/ma14247625)
Supplement: Supplementary file 1 [file materials-14-07625-s001.zip › materials-1469887-supplementary.pdf]

Table S1. Main traditional CI technologies [4-8].

Technology	Application
k-Nearest neighbors (k-NN)	They are used to map data into different classes based on specific requirements. The classification framework uses statistics that are not sensitive to, for example, application protocols.
Linear Discriminant Analysis (LDA)	
Support Vector Machine (SVM)	
Decision Trees	
Flow clustering using Expectation Maximization (EM)	Based on these features, the algorithm groups the data into a small number of clusters.
AutoClass	An unsupervised Bayesian classifier using expectation maximization (EM) algorithm to select best clusters from training dataset, whereby it repeats the EM search many times to reach a global maximum.
K-Means	Unsupervised ML uses partial feature information to capture negotiation phases that are different for different applications.
Density-based spatial clustering (DBSCAN)	It has the ability to classify noisy data unlike k-Means and AutoClass.
Fuzzy Logic Ordered Fuzzy Numbers (OFN)	It complements standard logic by giving the possibility to model under conditions that are imprecisely defined, when features are fuzzy subsets of a set of linguistic labels characterizing their outputs. Defuzzification methods allow the transformation of fuzzy outputs into crisp numbers. OFN allows the modelling of fuzzy processes in which direction matters.
Multifractal analysis	It is used to characterize the local regularity of a signal and the trend of its changes.

Table S2. Key challenges of CI technologies within 3D printing [3-7].

Area	Challenge
Feature engineering	Should be automated, especially if data generated by heterogeneous sources are noisy and exhibit non-trivial spatiotemporal patterns that require considerable effort and time to label.
Handling large amounts of data	Transmission of multidimensional geometric mobile data.
Labelled data requirements	Most current systems, including mobile, generate unlabeled or partially labelled data.
Data sharing	Single model can be trained for multiple targets without the need to completely re-train the model for different tasks, thus saving central processing unit (CPU) and portable memory.

Table S3. Traditional ANNs vs. DL [4-8].

	Traditional ANN	DL
Feature engineering	Require expensive feature engineering.	Automatically captures relationships from data with

		complex structure.
Handling large amounts of data	Training often requires storing all data, what is computationally infeasible in big data tasks.	Is able to handle large amounts of data and control over-fitting of the model, moreover it is adapted to a large number of different types of data generated at a fast pace. Models that are not scalable only require subsets of data at each training step.
Labelled data requirements	Is effective only if sufficient labelled data is available.	Some solutions are effective in dealing with unlabeled or partially labelled data.
Dedicated architectures	Layers in an ANN are rows of data points served by neurons using the same neural network. ANN uses weights and activation functions to learn, artificially replicating how the brain's neural network works. The weights are changed after each iteration by a neuron in the ANN. The ANN goes back and changes the weights depending on the accuracy calculated by the cost function. When it makes a mistake, it goes back and corrects the way it works.	They are effective in processing multi-dimensional geometric data or user location (including mobile) represented by coordinates, topology, metrics and sequence. CNN casts multiple layers on images and uses filtering to analyze the image input data. The purpose of these layers is to understand the patterns, process the output and provide an n-dimensional output vector. The aforementioned n-dimensional output is used to observe distinct features and combine them with the provided input image (e.g. for classification).
	Both methods use error measures to improve network learning and use epochs to analyze the performance of the generated models.	
